# Mesenchymal Stem Cell Infusion Shows Promise for Combating Coronavirus (COVID-19)- Induced Pneumonia

**DOI:** 10.14336/AD.2020.0301

**Published:** 2020-03-09

**Authors:** Ashok K Shetty

**Affiliations:** Institute for Regenerative Medicine, Department of Molecular and Cellular Medicine, Texas A&M University College of Medicine, College Station, Texas, USA

## Abstract

A new study published by the journal Aging & Disease reported that intravenous administration of clinical-grade human mesenchymal stem cells (MSCs) into patients with coronavirus disease 2019 (COVID-19) resulted in improved functional outcomes (*Leng et al., Aging Dis, 11:216-228, 2020*). This study demonstrated that intravenous infusion of MSCs is a safe and effective approach for treating patients with COVID-19 pneumonia, including elderly patients displaying severe pneumonia. COVID-19 is a severe acute respiratory illness caused by a new coronavirus named severe acute respiratory syndrome coronavirus 2 (SARS-CoV-2). Currently, treating COVID-19 patients, particularly those afflicted with severe pneumonia, is challenging as no specific drugs or vaccines against SARS-CoV-2 are available. Therefore, MSC therapy inhibiting the overactivation of the immune system and promoting endogenous repair by improving the lung microenvironment after the SARS-CoV-2 infection found in this study is striking. Additional studies in a larger cohort of patients are needed to validate this therapeutic intervention further, however.

A new study published by the journal “Aging & Disease” by a team led by Dr. Zhao reports that intravenous administration of clinical-grade human mesenchymal stem cells (MSCs) into seven patients with Coronavirus Disease 2019 (COVID-19) resulted in improved functional outcomes and facilitated recovery [1]. COVID-19 is a severe acute respiratory illness caused by a new coronavirus named severe acute respiratory syndrome coronavirus 2 (SARS-CoV-2) [2-3]. This new corona-virus has elicited a pandemic of respiratory ailment since December 2019. It first appeared in Wuhan, China, but has now disseminated to multiple countries in the world, including the United States [2-3]. Even with painstaking global restraint and confinement efforts, the prevalence of COVID-19 continues to climb, with an increasing number of new cases and significant mortality worldwide [3]. Coronaviruses are commonly found in people and multiple species of animals. Sometimes, animal coronaviruses infect people and spread from person to person [4]. SARS-CoV-2, one such example, causes mild to severe symptoms, which include fever, cough, and shortness of breath, but severe cases (~2%) have been observed to result in death [1-3].

The pathogenesis of SARS-CoV-2 has been suggested to include the recognition of the angiotensin I converting enzyme 2 receptor (ACE2) by its spike protein, and priming of its spike protein by the cellular transmembrane protease, serine 2 (TMPRSS2) facilitating host cell entry and spread [1,5-6]. Severe respiratory illness is a significant symptom of SARS-CoV-2 infection because the ACE2 receptor is widely expressed in the lung alveolar type II cells and capillary endothelial cells. Besides, alveolar cells express TMPRSS2 [1,7]. Infection of the lung by this virus leads to a cytokine storm with elevated levels of multiple proinflammatory cytokines, which causes edema, air exchange dysfunction, acute respiratory distress, secondary infection, which may result in death [1]. ACE2 expression is also seen in other tissues such as the heart, liver, kidney, and digestive organs. Such expression pattern explains why infected ICU patients are afflicted with not only acute respiratory distress syndrome but also other complications such as myocardial injury, arrhythmia, acute kidney injury, shock, and death from multiple organ dysfunction syndromes [1,8]. The World Health Organization has proclaimed this epidemic as a global public health emergency [3]. Currently, treating COVID-19 patients, particularly those afflicted with severe pneumonia, is challenging as no specific drugs or vaccines against SARS-CoV-2 are available [9]. Therefore, identifying a safe and effective treatment for severely affected COVID-19 patients is critical for saving lives.

In the study by Dr. Zhao and collaborators, seven patients with COVID-19 pneumonia displayed improved functional outcomes and recovered after an intravenous administration of clinical-grade human MSCs [1]. The chosen patients were positive for SARS-CoV-2, with one displaying critically severe type, 4 exhibiting severe types, and the other 2 showing common types of the syndrome. An additional three patients with severe types were enrolled for placebo control. Before MSC infusion, all patients displayed high fever, shortness of breath, low oxygen saturation, and pneumonia. The patients received 1 million MSCs per kilogram body weight when their symptoms were getting worse and were observed closely for 14 days. Notably, the study found that virtually all symptoms subsided by 2-4 days after receiving MSC infusion with no apparent adverse effects. Chest CT imaging demonstrated that chest pneumonia infiltration was significantly subsided. Also, the majority of patients showed negative results for the SARS-CoV-2 nucleic acid test over a week or two after MSC infusion. The overall improvement was quite extraordinary for an elderly patient in a critical condition after the infection [1].

MSCs have been employed extensively in cell therapy, which includes a plethora of preclinical research investigations as well as a significant number of clinical trials [10-13]. Safety and efficacy have been shown in many clinical trials. The notable examples include the immune-mediated inflammatory diseases, such as graft-versus-host disease and systemic lupus erythematosus [14-15]. Improved function after MSC infusions in multiple disease conditions has been mostly attributed to immunomodulatory effects, as these cells secrete a variety of paracrine factors, which interact with immune cells resulting eventually in immunomodulation [10-13]. The mechanisms underlying the improvements after MSC infusion in COVID-19 patients also appeared to be the robust antiinflammatory activity of MSCs. Such processes were evident from multiple beneficial outcomes, which include an increased number of peripheral lymphocytes, the decline in the C-reactive protein, and waning of overactivated cytokine-secreting immune cells (CXCR3^+^CD4^+^ T cells, CXCR3^+^CD8^+^ T cells, and CXCR3^+^ NK cells) by 3-6 days in the circulating blood [1]. Moreover, a group of CD14^+^CD11c^+^CD11bmid regulatory dendritic cell (DC) population increased after MSC treatment. Also, in comparison to the placebo group, the patients receiving MSCs displayed a decreased level of tumor necrosis factor-alpha, a major pro-inflammatory cytokine, with concurrent elevation in the concentration of the antiinflammatory protein interleukin-10 [1]. Furthermore, 10 x RNA-sequencing uncovered that infused MSCs were negative for ACE2 and TMPRSS2, which implied that MSCs were free from COVID-19 infection. Besides, multiple antiinflammatory and trophic factors were highly expressed in MSCs. Also, the Kyoto Encyclopedia of Genes and Genomes (KEGG) analysis suggested that MSCs were involved in antiviral pathways [1].

Remarkably, the study by Dr. Zhao and colleagues showed that intravenous MSC infusion could reduce the overactivation of the immune system and support repair by modulating the lung microenvironment after SARS-CoV-2 infection even in elderly patients. Intravenous infusion of MSCs typically leads to their accumulation in the lungs, where they secrete multiple paracrine factors [16]. Such factors likely played a significant role in protecting or rejuvenating alveolar epithelial cells, counteracting fibrosis, and improving lung function. MSC infusion would likely be particularly beneficial to elderly individuals infected with SARS-CoV-2, both with and without co-morbidities, as this population is more susceptible to SARS-CoV-2 induced pneumonia, resulting in severe respiratory distress and death because of immunosenescence [17-20]. In summary, this study showed that intravenous infusion of MSCs is a safe and efficient approach for treating patients with COVID-19 pneumonia, including in elderly patients displaying severe pneumonia. Additional studies in a larger cohort of patients are needed to validate this therapeutic intervention further, however.
